# Usage of nanobody-beta-galactosidase fusion in immunoassays and its application in detecting a peanut allergen

**DOI:** 10.1016/j.fochms.2026.100357

**Published:** 2026-01-18

**Authors:** Yuzhu Zhang, Shilpa R. Bhardwaj, Mathis Carrere, Xiaohua He, Tengchuan Jin, Yixiang Xu

**Affiliations:** a US Department of Agriculture, Agricultural Research Service, Pacific West Area, Western Regional Research Center, 800 Buchanan Street, Albany, CA 94710, USA; b Purpan Engineering School, 75, TOEC route - BP 57611, 31076 Toulouse, France^1^1 Permanent address.; c Division of Life Sciences and Medicine, University of Science and Technology of China, Hefei 230027, China

**Keywords:** Allergen detection, Enzyme immunoassay, ONPG, HRP, Nanobody-based ELISA

## Abstract

Small-sized nanobodies (NBs) offer many advantages over traditional antibodies and antibody fragments. β-Galactosidase (β-gal) has been used as a detection enzyme in immunoassays when horseradish peroxidase (HRP) or alkaline phosphatase (ALP) could not be used. However, more research is needed to fully realize the benefits of using β-gal in immunoassays. This study fused a previously isolated NB specific to peanut allergen Ara h 3 (Nb16) with the tetrameric *Escherichia coli* β-gal. Kinetic signals generated using ONPG demonstrated the advantage of using β-gal in ELISA experiments. Peanut allergen Ara h 3 was successfully detected with the Nb16-β-gal, with a detection limit of 0.3 ppm, outperforming detection with the same NB and HRP. For detecting peanut proteins in baked foods, the detection limit was better than 1.56 ppm. Stable signals produced with S-Gal/X-Gal showed the benefits of using β-gal in immunoblots. The readily available, stable β-gal substrates and the ease of recombinant production of NB-β-gal chimeras are among the advantages of using β-gal over HRP and ALP as detection enzymes in immunoassays.

## Highlights

• A fusion of *E.coli* β-gal and a nanobody against a peanut allergen was produced.
• The utility and advantages of β-gal in immunoassays were demonstrated.
• Low-cost substrates and stable signals showed β-gal's advantage over HRP and ALP.
• Easy production of Nb-β-gal chimeras signified their widespread use in the future.

## 1 Introduction

**Fig. 1:**
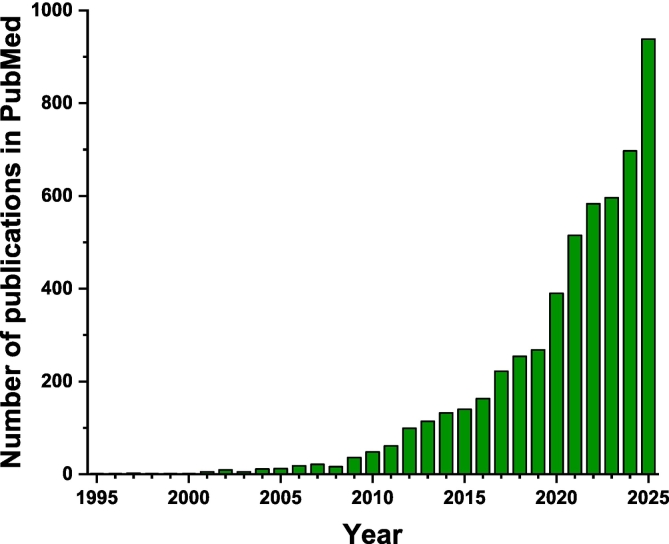
Growing research activity in nanobodies. A bar graph showing the number of research articles in the PubMed database (https://pubmed.ncbi.nlm.nih.gov) with titles or abstracts containing any of the following terms: single-domain antibody, nanobody, or nanobodies.

Heavy-chain-only antibodies (HCABs) in camelids (Hamers-Casterman et al., 1993) and cartilaginous fish were discovered three decades ago (Greenberg et al., 1995). The variable region of such an HCAB (VHH), also called a nanobody (NB), is the single domain responsible for antigen association. NBs have many desirable properties compared to conventional antibodies and antibody fragments, including small size, ease of production in *Escherichia coli* (*E. coli),* and thermal and conformational stability (van der Linden et al., 1999). These qualities make them valuable tools for research and applications in disease mechanisms, diagnosis, and therapy (Chen et al., 2023; Jovcevska & Muyldermans, 2020). The potential applications of NBs across numerous scientific fields have driven a surge of research over the past decade and a half (Fig. 1). NBs have also shown great promise in allergy research and treatment (Zettl, Bauernfeind, Kollarova, & Flicker, 2024). Recently, NBs targeting food allergens, including peanut allergen Ara h 3 (Chen et al., 2019), lupin allergen Lup an 1 (Hu et al., 2021), macadamia nut allergen Mac i 1 (Hu et al., 2021), milk allergen Bos d 5 (Hu et al., 2022), and crayfish allergen Pro c 2 (Yang et al., 2025), have been developed. NBs are also used in immunoassays for food safety applications to detect foodborne pathogens, pesticides, and mycotoxins (Hughes et al., 2023; Yang, Sun, He, Xie, & Liu, 2023; Zhang et al., 2019). NBs fused to a multimeric protein and alkaline phosphatase (ALP) were also engineered to reduce the steps in ELISA (Xie et al., 2022).

Immunoassays, including direct, indirect, sandwich, and competitive ELISAs, are among the most commonly used techniques for disease diagnosis and testing for foodborne contaminants (Liu, Chang, Lou, Zhang, & Yi, 2023). ALP and horseradish peroxidase (HRP) are the most commonly used reporter enzymes in immunoassays. Glucose oxidase and catalase have also been successfully used in immunoassays to generate signals for detection (Bari, Reis, & Nestorova, 2019; Ghavamipour et al., 2020; Wang et al., 2020). The easy large-scale production of small-sized single-domain antibody fragments derived from heavy-chain-only antibodies has made immunoassay reagents more accessible. This development opens the possibility of expanding the enzyme repertoire of these techniques.

β-Galactosidases (EC 3.2.1.23), commonly known as β-gals, are important glycoside hydrolases (GH, EC 3.2.1) found in animals, plants, and microorganisms. β-Gals are classified into four families: GH1, GH2, GH35, and GH42, but this classification has since expanded into eleven families (Zhou et al., 2024). The largest β-gal family is GH2, which is mainly produced by yeast and bacteria. An example of GH2 is *E. coli* β-gal. It is a tetrameric protein with identical subunits, each consisting of five structural domains plus an N-terminal extension of about 50 amino acids (AA) (Juers, Matthews, & Huber, 2012). A natural mutant missing a small part of its N-terminal region was inactive but could regain activity when complemented with peptides containing the missing N-terminal region (Juers et al., 2012). This phenomenon underlies the widely used blue-white screening in cloning. β-Gal is one of the most widely used enzymes in the food industry for producing lactose-free dairy products and lactulose with pharmaceutical applications (Liu, Chen, Ma, Ouyang, & Zheng, 2023). β-Gal was used as the detection enzyme in cell-ELISA to overcome the complications of using HRP or ALP, whose substrates could be hydrolyzed by the cells being assayed (Liu, Gurlo, & von Grafenstein, 2000). β-Gal was also used to reduce the steps in ELISA experiments (Anderson et al., 2018). Fusing the tetrameric β-gals with NBs also increases the NBs' valence. Given this well-characterized and widely used enzyme, we propose that, like HRP and ALP, β-gal can become a widely used detection enzyme in immunoassays. In this study, we aim to integrate NB-β-gal fusions into general-use immunoassay techniques. Peanuts are among the foods that cause the most severe food allergies. Previously, we obtained an NB specific to the peanut allergen Ara h 3 from a synthetic library (Chen et al., 2019). Here, we leverage this NB and the peanut allergen to develop NB-β-gal-based immunoassays.

## 2 Materials and methods

Milli-Q water was purified in-house using a Milli-Q Advantage A10 system (Millipore, Bedford, MA, USA) and used throughout. *o*-Nitrophenyl-β-galactoside (ONPG), Isopropyl β-D-1-thiogalactopyranoside (IPTG), Kanamycin (Kan), and X-Gal were purchased from GoldBio (St Louis, MO, USA). Stock solutions of IPTG and kanamycin at 1000× were prepared by dissolving the chemicals in Milli-Q water to 0.5 M and 50 mg/mL, respectively. A stock solution of X-Gal at 1000X was prepared by dissolving 20 mg of X-Gal in 1 mL of DMSO. S-Gal and ferric ammonium citrate were purchased from MilliporeSigma (St. Louis, MO, USA). A stock solution of S-Gal at 100× was prepared by dissolving 30 mg of S-Gal in 1 mL of water. The stock solutions were stored at −20 °C. Protein standards for gel filtration column calibration were obtained from GE Healthcare (Piscataway, NJ, USA). Prestained protein standards were purchased from Bio-Rad (Hercules, CA, USA). Other chemicals for cloning, bacterial growth, and protein purification were obtained from Fisher Scientific (Waltham, MA, USA). Ovalbumin, bovine serum albumin (BSA), and materials for protein characterization were from MilliporeSigma. Cow's milk allergen Bos d 4 (alpha-lactalbumin) was provided by Hilmar Cheese Company (Hilmar, CA, USA). All chemicals used in this study were of the highest grade available. Raw peanuts were purchased from nuts.com and stored at −20 °C before use. Commercial peanut butter (CPB) from three different manufacturers and other ingredients for preparing food matrices were purchased from local grocery stores.

### 2.1 Plasmid construction

A gene block encoding Nb16, an Ara h 3 specific NB isolated from a synthetic phage display library (Chen et al., 2019), was custom synthesized by IDT DNA Technologies (Coralville, IA, USA) with codon optimization, a seven-amino acid (AA) linker, and flanking cloning sites (*Afe* I at the 5′ end and *Pml* I at the 3′ end). There is a *Bam*H I site at the 5′ end within the linker. Additionally, adapter sequences ctcaaaactcaaaacagccat and gtgtctcaaaatctctgatgttac were present at the 5′ and 3′ ends of the gene block, respectively (Supplementary Material 1, Fig. S1). The gene block was PCR amplified with primers matching the adapter sequences and digested with *Afe* I and *Pml* I. The digested product was purified using an agarose gel and inserted into pTGS4 prepared with the same enzymes. pTGS4 is a homemade vector whose key properties have been described previously. The ligation product was used to transform chemically competent dH5α *E. coli*, and the transformants were spread on an LB plate containing kanamycin (LB/Kan plate). Five mL LB media containing kanamycin (LB/Kan) were inoculated with colonies for DNA miniprep using QIAprep Spin Miniprep Kits (QIAGEN Valencia, CA, USA) following the manufacturer's protocols. Positive clones carrying the Nb16 coding sequence in the correct orientation were identified by DNA sequencing performed by Elim Biopharm (Hayward, CA). The plasmid was named pRCH-Nb16GS.

The coding sequence of *E. coli* β-gal in the pcDNA3.1/*myc*-His(−)/*lac*Z vector (Invitrogen Carlsbad, CA, USA) was amplified by PCR using primers cgaatccacgtgatagatcccgtcgttttacaa (forward, with *Pml* I site) and cgaatcctcgagtttttgacaccagaccaact (reverse, with *Xho* I site). The PCR product was digested with *Pml* I and *Xho* I and ligated to the plasmid pTGS4 prepared with the same enzymes. After transforming dH5α *E. coli* with the ligation product, a positive colony was identified by DNA sequencing, and the resulting plasmid was named pT4-βGal, which harbors β-gal with a Strep-tag at its N-terminus and a His-tag at its C-terminus.

The β-gal coding sequence prepared with *Pml* I and *Xho* I was also ligated with the plasmid pRCH-Nb16GS prepared with the same enzymes. After transforming dH5α *E. coli* with the ligation product, a positive colony was identified through DNA sequencing, and the resulting plasmid was named pRCH-Nb16-βGal. This plasmid expresses Nb16 fused to a C-terminal His-tagged β-gal with a nine-amino acid (GSGASGSHV) linker between Nb16 and β-gal when induced in bacteria.

Primers gatcctggtcacaccctcaattcgagaaacac and gtgtttctcgaattgagggtgtgaccag were annealed together and ligated with the plasmid pRCH-Nb16-βGal, prepared with restriction enzymes *BamH* I and *Pml* I. After transforming dH5α *E. coli* with the ligation product, a positive colony was identified by DNA sequencing, and the resulting plasmid was named pRCH-Nb16-Tac-βGal, which expressed Nb16 fused to a C-terminal His-tagged β-gal with a twelve-amino acid (GSWSHPQFEKHV, which contained a strep-tap) linker between Nb16 and β-gal.

The PCR-amplified Nb16 gene segment was digested with *Afe* I and *Bam*H I, then ligated with the pT4-βGal plasmid prepared with the same enzymes. After transforming dH5α *E. coli* with the ligation product, a positive colony was identified via DNA sequencing, and the resulting plasmid was named pRCH-Nb16βGal, which expressed Nb16 fused to a C-terminal His-tagged β-gal, with a four-amino acid (GSHV) linker between Nb16 and β-gal.

### 2.2 Expression of β-gal-tagged Nb16

Competent *E. coli* BL21 (BL21 Star™ (DE3), Fisher Scientific) was transformed with each of the plasmids described above. A colony was selected to start an overnight 50 mL LB/Kan culture at 37 °C in an incubator with shaking at 200 rpm. One-liter cultures were started by adding 25 mL of the overnight culture to 950 mL of LB/Kan media. When the OD at 600 nm of the cultures reached approximately 1, the shaker temperature was lowered to 18 °C, and the expression of β-gal-tagged Nb16 was induced one hour later by adding IPTG. The bacteria were harvested the next day (after 14–18 h of incubation) by centrifugation at 3900 *g*; all centrifugation was carried out at room temperature (∼20 °C) unless otherwise stated. The pellets were resuspended in a His-binding buffer (Buffer HB, 20 mM imidazole, pH 8.0, 500 mM NaCl) and stored at −20 °C.

### 2.3 Nb16-β-gal purification

The frozen cell suspension was thawed by placing the tube in a beaker filled with tap water. The suspension was diluted to 65 mL with Buffer HB and sonicated after adding benzamidine to a final concentration of 1 mM. The sample was centrifuged at 18 °C and 20,000 *g* for 40 min. Nb16-β-gal fusions were purified using two chromatography steps with an AKTA FPLC system (GE Healthcare). For each fusion variant, the cleared lysate was loaded into a 5-mL HisTrap FF Crude column (GE Healthcare) pre-equilibrated with 25 mL of Buffer HB. Unbound proteins were washed with 25 mL of Buffer HB and 25 mL of a His-wash buffer (45 mM Imidazole, pH 8.0, 500 mM NaCl). The fusion protein was eluted with a His-elution buffer (300 mM Imidazole, pH 8.0, 500 mM NaCl) and further purified using size-exclusion chromatography (SEC) on an XK 26/70 column packed with prep-grade Superdex 200 (GE Healthcare). The column (with a bed volume of 344 mL) was pre-equilibrated and run with Buffer B (10 mM Tris, pH 8.0, 200 mM NaCl).

### 2.4 Purification of peanut allergen Ara h 3

The purification of the major peanut allergen Ara h 3 was performed using previously described methods with minor modifications (Chen et al., 2019; Jin, Howard, & Zhang, 2007). Briefly, 15 g of dry peanut kernels were ground in a KitchenAid blender with 50 mL of Buffer B and 50 mL of hexane. The ground sample was centrifuged at 3500 *g* for 10 min, and the middle layer was collected as a crude extract. The crude extract was mixed with 0.37 volumes of a saturated ammonium sulfate solution (4.06 M) and centrifuged at 16,700*g* for 40 min. The supernatant was collected and combined with 0.46 volumes of saturated ammonium sulfate solution. The mixture was centrifuged again at 3500*g* for 10 min, and the pellet was resuspended in 50 mL of Buffer A (10 mM Tris, pH 8.0). The sample was filtered through 0.45 μm pore-size filters and loaded onto an 8-mL Mono Q column (GE Healthcare) pre-equilibrated with Buffer A, then eluted with Buffer A and a linear NaCl gradient from 0 to 1 M over 120 mL. Column purification was performed using an AKTA FPLC system (GE Healthcare). Fractions containing Ara h 3 were pooled, and ammonium sulfate was added to reach 19.8% (w/v). The sample was filtered through 0.45 μm pore-sized filters and loaded onto a 5 mL HiTrap Phenyl FF column (GE Healthcare) pre-equilibrated with Buffer A containing 19.8% (w/v) of ammonium sulfate. Bound proteins were eluted with a gradient of ammonium sulfate from 19.8% to 0% in Buffer A. Fractions containing Ara h 3 were pooled, and an additional 53% (w/v) of ammonium sulfate was added to the sample. The sample was centrifuged at 3500 *g*, and the pellet was resuspended in Buffer B, filtered through a 0.45 μm filter, and loaded onto the Superdex 200 column (GE Healthcare) pre-equilibrated with Buffer B, then eluted with Buffer B. The purified protein was concentrated to 1 mg/mL, aliquoted, and flash-frozen in liquid nitrogen, then stored at −80 °C for further use.

### 2.5 Sodium dodecyl sulfate-polyacrylamide gel electrophoresis (SDS-PAGE) analyses

For Nb16-β-gal induction test samples, 500 μL of both uninduced and induced bacteria were collected by centrifugation at 2400 *g* using a microcentrifuge. The pellets were resuspended in 50 μL of 8 M urea, mixed with 50 μL of a 2× SDS sample buffer (50 mM Tris-HCl, pH 6.8, 2% SDS, 0.1% bromophenol blue, 10% glycerol) containing 100 mM β-mercaptoethanol (β-Me), and incubated at 96 °C for 10 min. Purified Ara h 3 was mixed 1:1 with the 2× SDS sample buffer, with and without β-Me for reduced and non-reduced samples, respectively. Control samples of α-lactalbumin and ovalbumin were prepared similarly for SDS-PAGE. SDS-PAGE was performed using Invitrogen Bolt Bis-Tris Plus mini protein gels (4–12%) with a Bolt MES SDS running buffer (Fisher Scientific). Gels were stained with Coomassie Brilliant Blue (CBB) using previously described protocols (Zhang et al., 2014), and the results were documented with an ImageQuant LAS 400 Imager (GE Healthcare).

### 2.6 Food sample preparation

Peanut muffins were prepared following a published protocol (Nelis, Broadbent, Bose, Anderson, & Colgrave, 2022) with minor modifications. Briefly, 60 g of unbleached wheat flour, 60 g of milk, 30 g of CPB, 15 g of sugar, 10.5 g of unsalted butter, 10 g of whisked raw egg, 2.5 g of baking soda, and 0.05 g of table salt were mixed. The muffins were baked at 180 °C for 20 min. After cooling, the muffins were weighed, and the peanut protein content was calculated using the manufacturer's reported peanut protein content for CPB (7 g per 32 g) and the muffins' total weight. A blank matrix was prepared by making muffins without CPB. The moisture content of the muffins was determined using an MX-50 moisture analyzer (A&D company, Ann Arbor, MI, USA).

100 mL of TBS was added to 10 g of the muffins. The samples were vortexed and then sonicated for 6 min. To remove fat, 50 mL of hexane was added, and the mixture was centrifuged at 4000*g* for 15 min. The aqueous phase was collected and then centrifuged at 15 °C and 20,000*g* for 40 min. The middle portion of the clear samples was filtered through 0.22 μm filters, aliquoted, and stored at −80 °C.

### 2.7 ELISA

Ninety-six-well black plates with untreated transparent surfaces (Thermo Fisher Scientific, Waltham, MA, USA) were coated overnight at 4 °C with 100 μL of the specified proteins in TBS (25 mM Tris, pH 7.4, 137 mM NaCl, 2.7 mM KCl, 0.1%) at the designated concentrations. After coating, the plates were washed three times with TBST (TBS containing 0.1% Tween 200) and twice with TBS, with each wash lasting 5 min at room temperature. The plates were then blocked with 150 μL of TBS containing 3% (w/v) BSA for one hour, followed by three washes with TBST and two with TBS. Next, the plates were incubated with 100 μL of TBS containing 5 μg/mL Nb16-β-gal and 0.3% (w/v) BSA for 60 min. The plates were washed four times with TBST and twice with TBS. For detection, 100 μL of a lacZ-β-gal substrate buffer (Buffer Z: 100 mM sodium phosphate, pH 7.0, 10 mM KCl, 1 mM MgSO_4_) containing 1 mg/mL (w/v) ONPG was added to each well. The absorbance at 420 nm was measured with a Sunrise plate reader (Tecan, Morrisville, NC, USA) for 60 min at 30-s intervals.

Similar experiments were conducted to evaluate whether NB-β-gal fusions can be used in direct ELISA tests to detect allergens in food samples.

### 2.8 Statistics

To analyze the ELISA results, signals from the triplicate wells were averaged, and the mean was plotted against time to generate kinetic curves. Linear fitting of the kinetic data was performed using OriginPro (OriginLab Corp, Northampton, MA, USA). The average signals obtained at 60 min were used to create the endpoint bar graphs. All immunoassays were carried out independently three times to ensure the data can be reproduced. CPBs from three manufacturers were used to prepare different food samples, ensuring the results were not affected by the ingredients or sample preparation.

### 2.9 Immunoblots

For dot blot experiments, a polyvinylidene fluoride (PVDF) membrane was spotted with 2 μL of a 200 μg/mL Ara h 3 and Bos d 4. Additionally, 1 μL and 0.5 μL drops of the Ara h 3 sample were also spotted on the PVDF membrane. The membranes were blocked with 10% nonfat dry milk (w/v) in TBS for one hour at room temperature. The blocked membranes were incubated with Nb16-β-gal diluted to 5 μg/mL in TBS containing 1% nonfat milk for 60 min. The membranes were washed three times with TBST for 5 min each, followed by two washes with TBS. Then, the membranes were incubated with Buffer Z containing 0.03% S-Gal (w/v) and 0.05% (w/v) ferric ammonium citrate. The experimental results were photographed with a cell phone at different times.

## 3 Results and discussions

### 3.1 Fusing NBs to β-gal

With the rapid increase in the number of available NBs targeting various molecules of interest to scientists across different research fields, it is understood that limited valence of NBs can be a drawback in some applications. Different methods have been used to achieve multivalence (Glassman et al., 2020; Hesen et al., 2024; Hughes et al., 2023). One of these is to fuse NBs with multimeric proteins (Liao et al., 2024). Bacterial β-gal is a tetrameric protein, and its easy production makes it a good candidate for fusion with NBs. The enzymatic activity of β-gal has been utilized in industries to produce commodity products and in academia to accelerate research. We sought to utilize β-gal activity in colorimetric immunoassays to assess NB-antigen association while using β-gal's multimeric property to increase the valence of NBs.

**Fig. 2:**
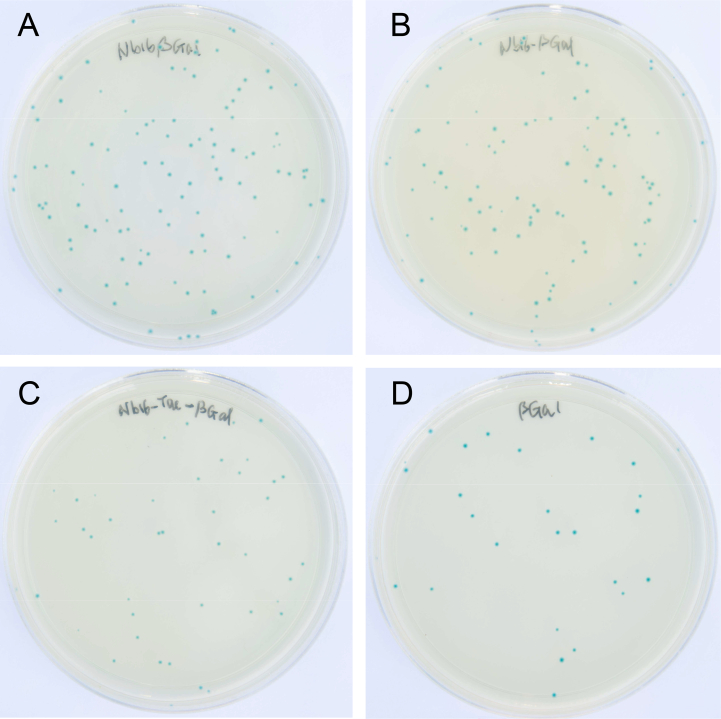
Tagging a nanobody to the N-terminus of β-gal does not affect its activity. (A) Colonies of dH5α bacteria transformed with a plasmid containing the coding sequence for β-gal with Nb16 fused to its N-terminus via a 4AA (GSHV) linker and plated on an LB/Kan plate with IPTG and X-gal. The plate was photographed 24 h after incubation at 37 °C for 16 h. (B), (C), and (D) are the same as (A), except that the bacteria were induced to express β-gal with Nb16 fused to its N-terminus via a longer flexible peptide (GSGASGSHV), a Strep-tag-containing peptide (GSWSHPQFEKHV), or β-gal with purification tags, respectively.

To determine whether attaching an NB to β-gal with different linkers affects β-gal activity, dH5α bacteria harboring pT4-βGal, pRCH-Nb16βGal, pRCH-Nb16-βGal, or pRCH-Nb16-Tac-βGal were plated on LB agar with kanamycin, IPTG, and X-Gal. The color development of the colonies was photographed, and the images are shown in Fig. 2. The results showed that dH5α expressing Nb16 fusion of β-gal with four amino acids between the two proteins, encoded by the enzyme sites for cloning (Nb16-β-gal, Fig. 2A), dH5α producing Nb16-β-gal fusion with a nine-AA linker connecting the two proteins (Fig. 2B), and dH5α producing a fusion with a twelve-AA linker connecting the two proteins (Nb16-Tac-β-gal, Fig. 2C), all developed a blue color as intense as the blue color developed in the colonies of dH5α with pT4-βGal, which produced β-gal with only short Step- and His-tags for purification (Fig. 2D). These data indicated that attaching an NB to the N-terminus of β-gal with the designed linkers did not significantly affect the activity of β-gal. In the experiments described below, the Nb16 β-gal fusion with a 9-AA linker (Nb16-β-gal) was used to test whether β-gal can facilitate the detection of antibody-antigen associations by ELISA and immunoblot experiments.

### 3.2 Expression and purification of Nb16-β-gal

**Fig. 3:**
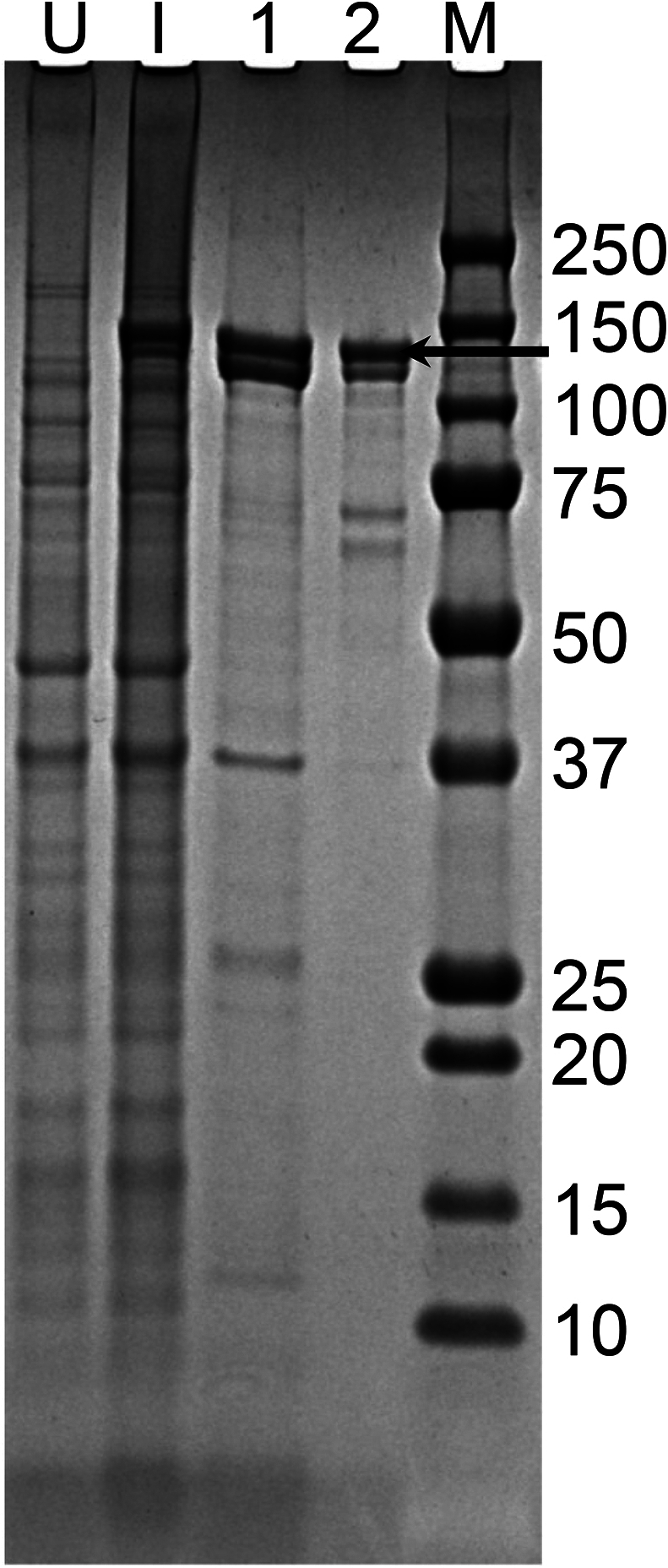
Expression and purification of Nb16-β-gal. BL21 bacteria carrying pRCH-Nb16-βGal were used to produce the target protein. Proteins in lysates from uninduced (lane U) and induced (lane I) bacteria, fractions containing the target protein after Ni^2+^ column purification (lane 1), and the purified target protein after the second-step SEC purification (lane 2) were analyzed by SDS-PAGE on a 4–12% SDS gel. The molecular masses (in kDa) of the proteins in the protein standards (lane M) are indicated on the right side of the gel image. The horizontal arrow indicates the Nb16-β-gal band.

Bacteria (such as dH5α) with inactive β-gal are widely used to select positive clones during plasmid construction in molecular biology because inactive β-gal can regain its function when complemented with an α-component. Plasmids containing a sequence encoding functional β-gal are often used as positive controls or reporters when β-gal is only expressed under specific conditions (Zhang et al., 1999). In this study, we amplified the β-gal coding sequence from the positive control plasmid pcDNA3.1/myc-His(−)/lacZ and constructed pRCH-Nb16-βGal. When expressed in BL21 bacteria, the Nb16-β-gal protein was a C-terminal poly-histidine-tagged fusion. Most of the expressed fusion protein was recovered in the supernatant after freezing, thawing, sonication, and centrifugation. After a one-step purification using a HisTrap column in immobilized metal affinity chromatography, the sample showed high purity but still contained several impurities (Fig. 3). We further purified the protein using a Superdex 200 column (Fig. 3), and the two-step-purified protein was used to develop NB-β-gal-based immunoassays.

### 3.3 Purification of Ara h 3

**Fig. 4:**
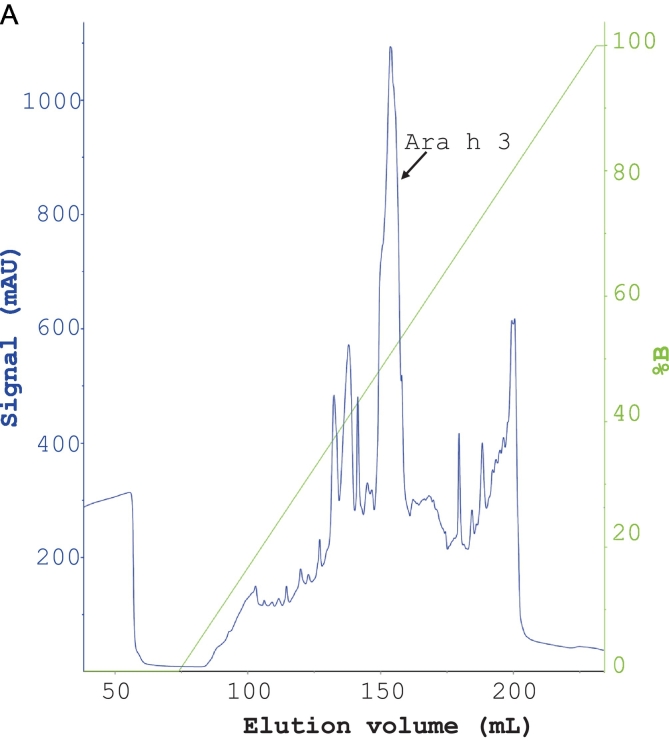

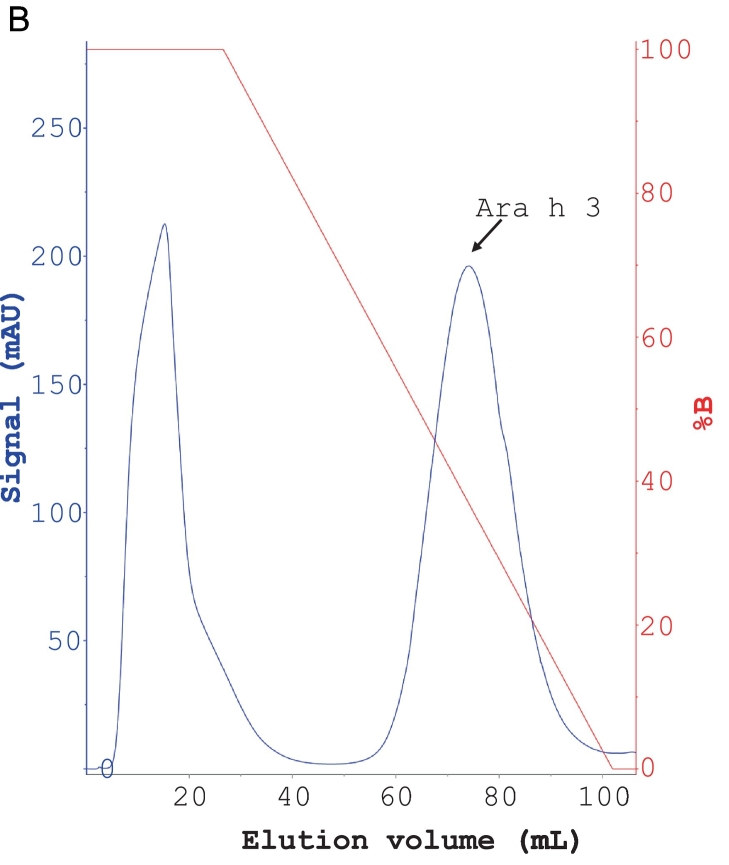

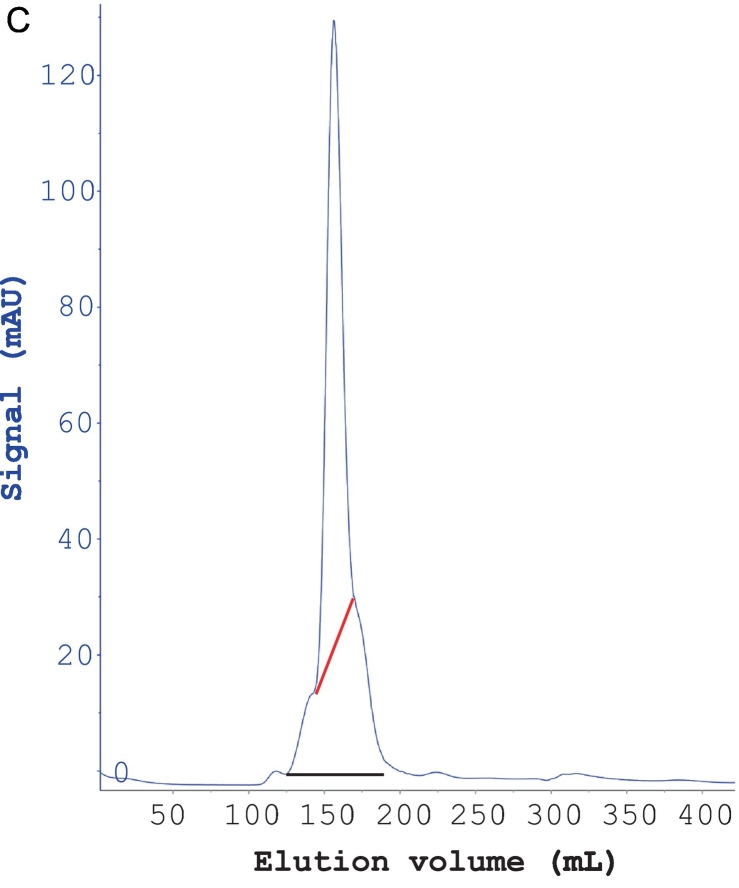

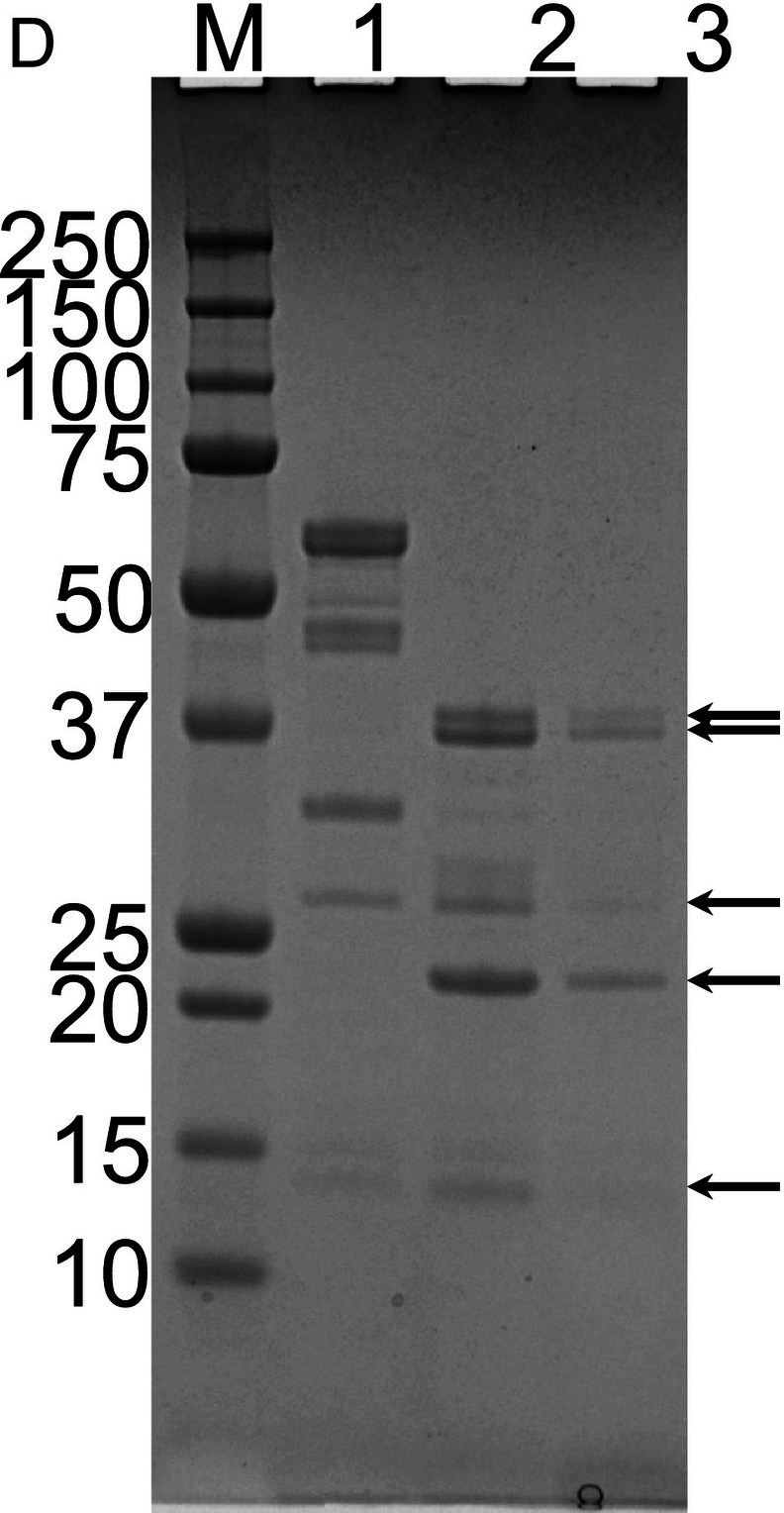
Purification of Ara h 3. (A) Anion exchange purification of Ara h 3 obtained from crude peanut extract by ammonium sulfate precipitation. (B) Hydrophobic chromatography of Ara h 3 following anion exchange. (C) SEC purification of Ara h 3 following HIC. (D) SDS-PAGE analysis of purified Ara h 3. Nonreduced (lane 1) and reduced (lane 2) Ara h 3 samples were separated on a 4–12% gel. As previously reported, Ara h 3 resolved into multiple bands indicated by arrows. Reduced Ara h 3-S (lane 3) (see text) was analyzed alongside Ara h 3. The molecular masses (in kDa) of the proteins in the marker (lane M) are shown on the right side of the gel images.

In this study, we replaced the dialysis step in a previously established protocol for purifying Ara h 3 with stepwise ammonium sulfate precipitations. This modification makes the method less time-consuming. The chromatographic purifications of Ara h 3 are shown in Fig. 4A–C. In the SEC step, Ara h 3 eluted as a peak with small shoulders, with the front edge at 145 mL. The estimated molecular mass of the main peak was approximately 347 kDa based on column calibration, indicating that Ara h 3 is a hexamer in solution, consistent with previous reports (Chen et al., 2019; Jin, Guo, Chen, Howard, & Zhang, 2009). The fractions from the central part of the peak (shown by the red line in Fig. 4C) were pooled as Ara h 3, while fractions from the small shoulders (fractions covered by the black line under the peak, excluding those pooled as Ara h 3) were collected as Ara h 3-S. SDS-PAGE analysis showed that Ara h 3 and Ara h 3-S had similar band patterns (Fig. 4D), indicating that the purified protein is not a single peptide chain, consistent with previously reported results for Ara h 3. The Ara h 3 purified in this study is similar to that purified by Chen et al. (Chen et al., 2019) but showed more bands than the sample used for crystallization studies (Jin et al., 2007), suggesting that the purified samples may contain more than one isoform of Ara h 3. The Ara h 3 from the central part of the SEC purification was used in immunoassays.

### 3.4 Using β-gal to generate signals in ELISA

**Fig. 5:**
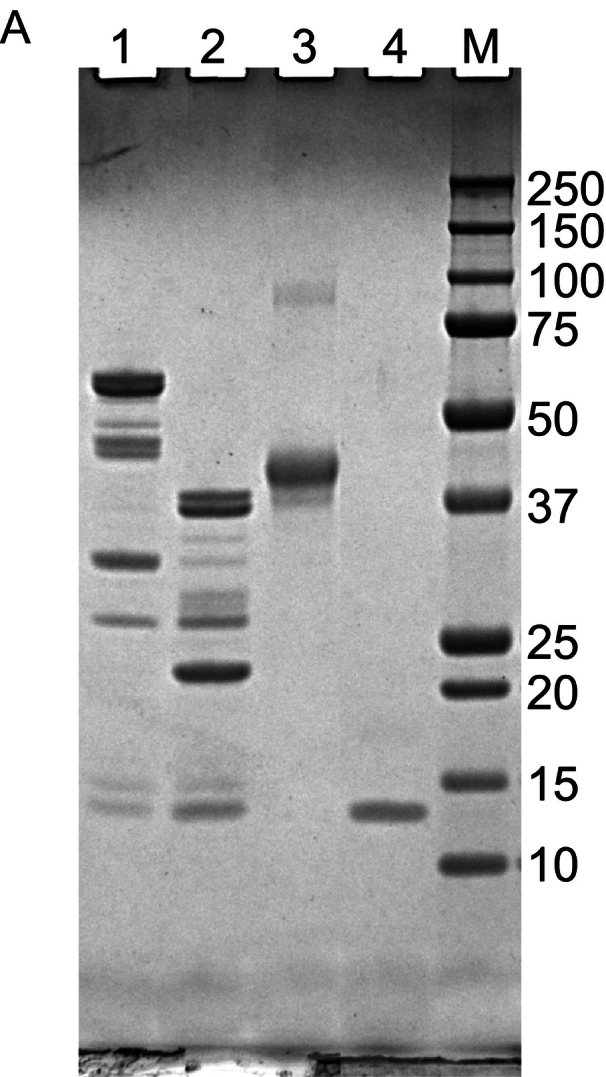

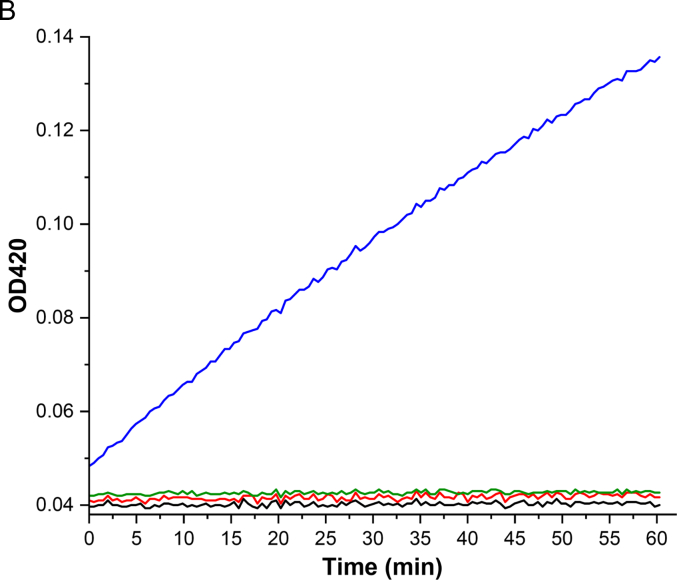

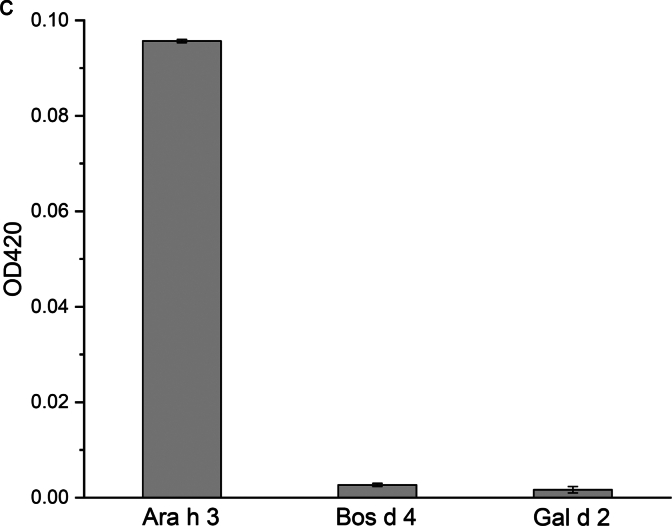
β-Gal detection of the association between its fusion partner Nb16 and Ara h 3 by ELISA. The utility of β-gal as a colorimetric enzyme in ELISA was assessed. Nb16-βgal association with Ara h 3 coated on the surface of the wells, but not with the control proteins, was detected. (A) SDS-PAGE analysis of Ara h 3 and control proteins used to coat the microplate. Nonreduced (lane 1) and reduced (lane 2) Ara h 3 were separated on a 4–12% SDS gel and stained with CBB. Chicken allergen Gal d 2 (lane 3) and cow's milk allergen Bos d 4 (lane 4) were included as control samples. The molecular masses (in kDa) of the proteins in the marker (lane M) are shown on the right side of the gel images. (B) The kinetic curves of the signal readout during plate incubation after the β-gal substrate ONPG was added. The black line shows the average signal of the wells incubated with TBS during the coating step. Red, green, and blue lines show the average signals of the wells coated with Gal d 2, Bos d 4, and Ara h 3, respectively. All the coating samples were at a concentration of 20 μg/mL. (C) A bar representation of β-gal detection in the ELISA experiment using the endpoint data. (For interpretation of the references to color in this figure legend, the reader is referred to the web version of this article.)

To assess whether β-gal fused with an NB can be used in ELISA, Ara h 3 and the control proteins shown in Fig. 5A were coated onto 96-well plates in triplicate overnight at 4 °C. After blocking and incubation with the β-gal fused NB, the absorbance at 420 nm was measured following the addition of ONPG in Buffer Z. The data are presented in Fig. 5B. Additionally, the ELISA results are presented as a bar graph using endpoint absorbance values (Fig. 5C). Comparing the signals from wells coated with Ara h 3, ovalbumin, and α-lactalbumin, it can be concluded that β-gal can reliably detect antibody-antigen interactions, which also simplifies the ELISA experiments by reducing the number of steps.

**Fig. 6:**
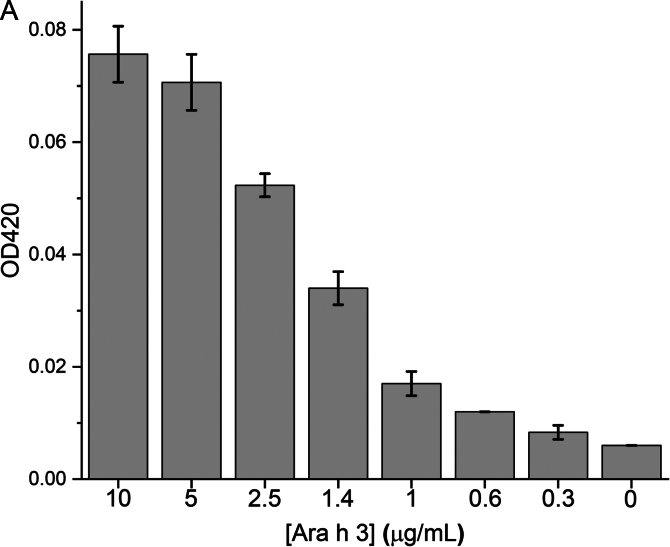

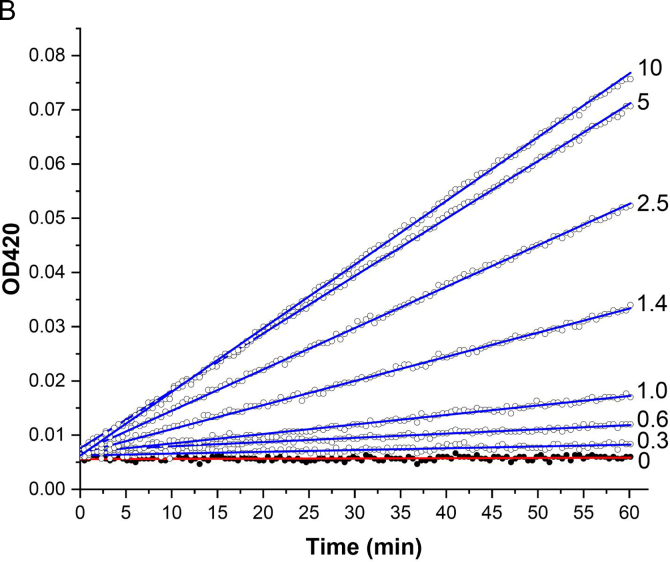

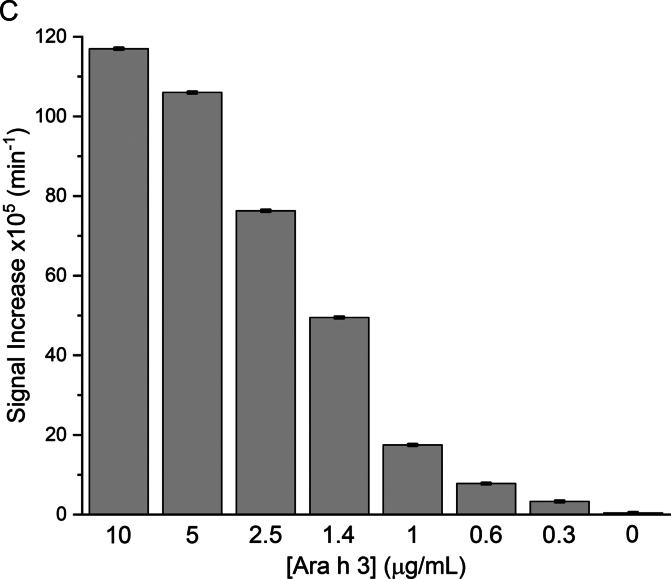

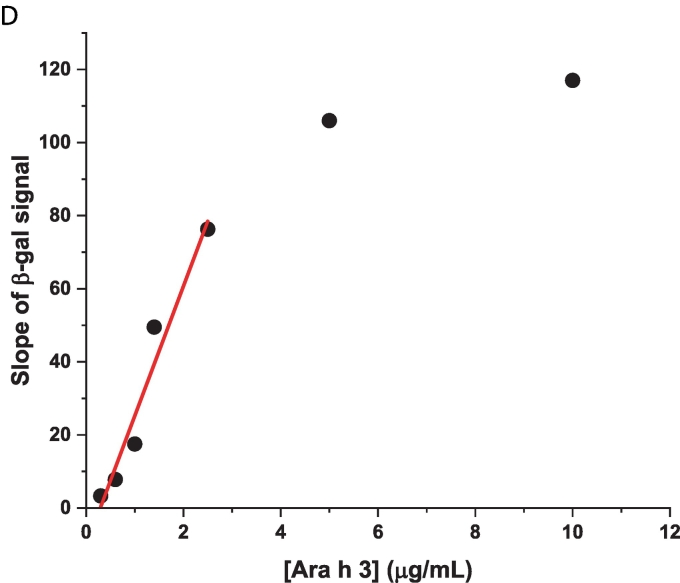

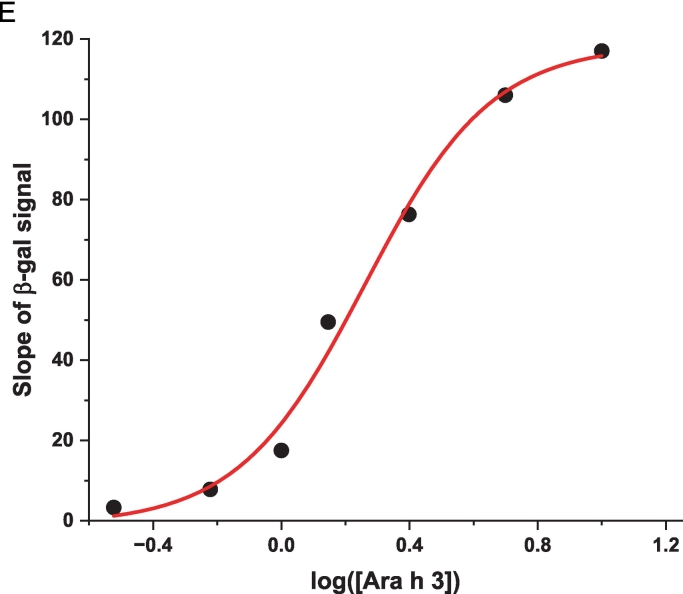
Detection of peanut allergen Ara h 3 at various concentrations. (A) Plate wells were coated with Ara h 3 at the concentrations indicated below the bar plot. Results were analyzed as described in Fig. 5C, but without background (signal for [Ara h 3] = 0) correction. (B) Kinetic signal readout during plate incubation after the β-gal substrate ONPG was added. Ara h 3 concentrations are indicated next to the endpoint signals. (C) The slopes of the linear fit to the kinetic data are shown in a bar graph. The Ara h 3 concentrations in the coating samples are indicated under the bars. (D) The slope of the kinetic data as a function of the Ara h 3 concentration is shown. The red straight line shows the results of fitting the data for Ara h 3 concentrations <2.5 μg/mL. (E) A semi-log plot of the slopes of the β-gal signal against the Ara h 3 concentration. The red sigmoidal line shows the result of a four-parameter logistic curve fit. (For interpretation of the references to color in this figure legend, the reader is referred to the web version of this article.)

To demonstrate that Nb16-β-gal can detect Ara h 3 in a dose-responsive manner in the β-gal-based colorimetric ELISA, serial dilutions of Ara h 3 were prepared and used to coat triplicate wells of a 96-well plate. After coating, blocking, and washing, the plate was incubated with Nb16-β-gal. ONPG was added after unbound Nb16-β-gal was removed by washing. The ELISA absorbance signal at 420 nm was recorded over 60 min using a plate reader. The endpoint bar graph of results is shown in Fig. 6A**,** and the ELISA kinetic curve is shown in Fig. 6B. The endpoint bar graph shows that the signal for the sample at the lowest Ara h 3 concentration tested (0.3 μg/mL) is less than twice that of the background controls. However, Fig. 6B indicates that the kinetic data are more convincing that Nb16-β-gal could detect Ara h 3 at the given concentration, demonstrating the advantage of analyzing kinetic data with many data points over an endpoint experiment that relies on a single data point. With kinetic data, the rate of signal increase can be used to assess the presence of the antigen. The slopes of the linear fits to the kinetic data and their standard errors are shown in Table 1. A bar graph was constructed using the slopes of the kinetic curves (Fig. 6C). The benefit of collecting kinetic data is readily apparent in comparing Fig. 6A and Fig. 6C. The rate of signal increase as a function of the allergen concentration is shown in Fig. 6D. It can be concluded from Fig. 6C and D that the ELISA detection limit is at least 0.3 μg/mL, which is better than that obtained with a direct ELISA using phage ELISA and assayed using HRP as the detection enzyme (Chen et al., 2019). Note that Nb16 was isolated from a synthetic phage library with a binding affinity of 400 nM to Ara h 3. When NBs with higher affinity are obtained, using the strategy presented here, an ELISA kit with higher sensitivity can be constructed. The results are also presented in a semi-logarithmic plot (Fig. 6E). The data indicated that NB-β-gal fusions can also be used for quantitative ELISA when the amount of a specific antigen present in a sample is desired.

**Table 1:** ELISA signal changes per minute based on the linear fitting of the kinetic data.

[Ara h 3] (μg/mL)	10	5	2.5	1.4	1.0	0.6	0.3	0
Slope (min^−1^)	1.17E-3	1.06E-3	7.63E-4	4.95E-4	1.75E-4	7.8E-5	3.3E-5	4 E-6
Standard error	2.3E-6	2.1E-6	2.3E-6	2.1E-6	2.0E-6	1.6E-6	1.9E-6	1.8E-6

Using HRP as the detection enzyme with currently available HRP substrates only allows endpoint ELISA assays. Compared to ALP as the detection enzyme in ELISA experiments, using β-gal involves reagents that are less chemically hazardous. Therefore, these data suggest that β-gal can be used in ELISA to generate detection signals, offering some advantages over HRP and ALP, in addition to the ease of producing NB-β-gal fusions.

### 3.5 Using β-gal to generate signals in immunoblots

**Fig. 7:**
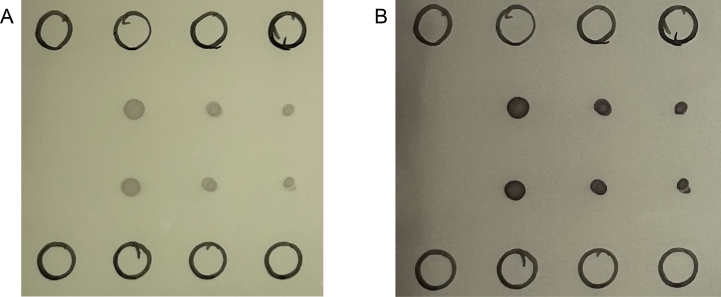
Detection specificity of Nb16 by immunoblot. The utility of β-gal as an enzyme for colorimetric detection in immunoblot was assessed. Black circles were drawn with a permanent marker to provide coordinates for spotting proteins onto the membrane. Control sample Bos d 4 was spotted at rows 2 and 3 (counting from the top) in the first column (counting from the left). Ara h 3 was spotted at rows 2 and 3 of the second column. Ara h 3 was also spotted at columns 3 and 4, with ½ and ¼ of the sample volume spotted at column 2.

To evaluate the usefulness of NB-β-gal fusions in immunoblots, Ara h 3 and Bos d 4 were applied to a PVDF membrane, and Nb16-β-gal was used to detect Ara h 3. Fig. 7 shows the results when S-Gal was used as the β-gal substrate. The data suggest that β-gal is effective in immunoblot experiments. Because β-gal catalyzes the conversion of part of S-Gal into a stable, insoluble product with a color indication, β-gal is advantageous over HRP, which catalyzes a reaction that converts the currently available substrate to give a short-lived signal for luminol-based substrate or a signal only when the reaction is stopped by changing the pH of the reaction mixture in the case of TMB (3,3′,5,5′-Tetramethylbenzidine)-based detection. Note that X-gal can also be used as the β-gal substrate in immunoblot experiments, though the signal develops more slowly than when S-Gal was used (data not shown).

### 3.6 β-Gal can be used to detect allergens in processed food

**Fig. 8:**
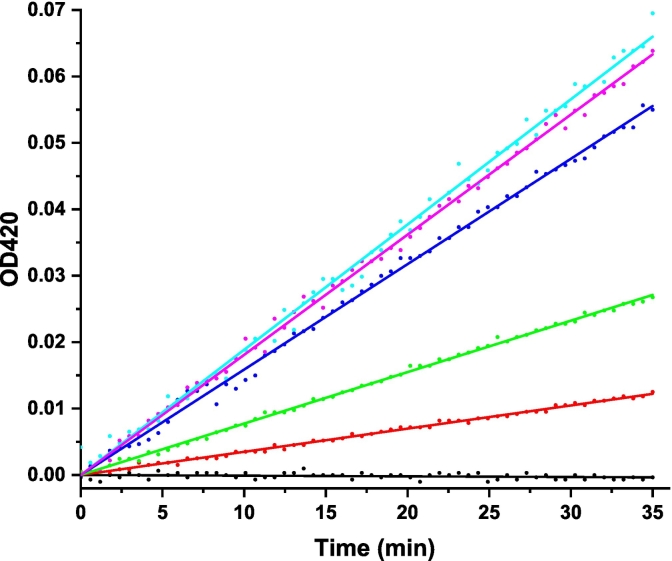
Direct ELISA detection of peanut proteins in baked food. Kinetic signal readout during plate incubation with β-gal substrate ONPG. Each data point is the average of three triplicate wells. Data obtained by coating the plate with diluted muffin extract at peanut protein concentrations of 1.56, 3.13, 6.25, 15.63, and 39.06 ppm are shown in red, green, blue, cyan, and magenta, respectively. Data for the negative control, with wells treated with TBS during coating, are shown in black. Linear fits were applied to each data set, and the y-axis intercept of each fit was subtracted from each data point to shift the data set vertically. The straight lines are the results of linear fits of the shifted data. (For interpretation of the references to color in this figure legend, the reader is referred to the web version of this article.)

Because β-gal is commonly present in microbes (bacteria, fungi, yeast), plants, and animals (Zhou et al., 2024), its presence in test samples complicates its use in direct ELISA. Baked goods are the most frequently recalled food type in the FDA Reportable Food Registry for undeclared allergens (Parker et al., 2015). We hypothesized that in packaged processed foods, naturally occurring β-gal is denatured and loses its function. We aimed to determine whether the Nb16-β-gal-based direct ELISA can detect peanut allergens in muffins. The results of a typical ELISA experiment using Nb16-β-gal to detect peanut protein in the muffins are shown in Fig. 8 and Table 2. Results of more independent ELISA assays detecting peanut proteins in muffins prepared using CPB from different manufacturers are shown in Supplementary Material 2, Figs. S3–S5. These data indicate that the method can detect peanuts in baked goods, with a limit of detection of 1.56 ppm or better.

**Table 2:** Detecting peanut protein in a food by ELISA with NB-β-gal.

[peanut protein] (ppm)	39.06	15.63	6.25	3.13	1.56
Slope (min^−1^)	1.81E-3	1.89E-3	1.59E-3	7.74E-4	3.49E-4
Standard error	1.3E-5	1.9E-5	1.2E-5	4.4E-6	3.5E-6

In addition, if any β-gal is present in the food matrices, it will be removed during the washing step after the allergen binds to the capture antibody in sandwich ELISAs. Therefore, the potential presence of native β-gal in a test sample that is not denatured by food processing can be assessed using a sandwich ELISA.

Although mass spectrometry has been considered an alternative method for food allergen detection over the past decade, ELISA, particularly sandwich ELISA, has remained the method of choice (Amado, Pazos, & Carrera, 2025; Nelis et al., 2022; Senyuva, Jones, Sykes, & Baumgartner, 2019).

Generally, it remains true that the recombinant production of HRP has not been very successful (Patmawati et al., 2018). As a result, this study demonstrates that stable, inexpensive substrates make β-gal an advantageous enzyme for generating stable signals in immunoassays.

The available cloning sites in the plasmids generated in this study can also be used to make β-gal fusions with other NBs by replacing the coding sequence of Nb16 (Supplementary Material 1, Fig. S2) with sequences encoding the NBs of interest. Additionally, if needed, the linker between the NB and β-gal can be modified using annealed primers. Thus, this strategy can be easily adapted to incorporate NBs into a wide range of immunoassays. As more NBs specific to molecules of importance in various research, industry, and healthcare fields are developed, the ease of expressing and purifying NB-β-gal fusion proteins accelerates reagent production. β-Gals from other β-gal families can have monomeric (Rico-Diaz et al., 2017), dimeric (Kil et al., 2024), and trimeric structures (Fan et al., 2016). Cold-adapted and thermostable β-gals have been isolated from psychrophilic and thermophilic microorganisms (Fan et al., 2015; Murphy & Walsh, 2019). The industry applications of β-gal have driven research to discover new β-gals and enhance existing ones through protein engineering. Such research will also help advance future immunoassays that utilize β-gal.

## 4 Conclusions

A β-gal fusion with an NB specific for the peanut allergen Ara h 3 at its N-terminus was recombinantly expressed and purified. The NB-β-gal fusion was used to detect Ara h 3 by ELISA and immunoblot. All reagents used in this study are commercially available or easily reproducible. When NBs with higher affinities for additional food allergens are developed, producing new β-gal fusions with NBs will be straightforward. These materials can be used to create standardized ELISA kits for processing facilities to test for the possible presence of peanuts in food products due to cross-contamination, thereby reducing the likelihood of costly food recalls and potentially saving lives.

## CRediT authorship contribution statement

**Yuzhu Zhang:** Writing – review & editing, Writing – original draft, Supervision, Methodology, Conceptualization. **Shilpa R. Bhardwaj:** Writing – review & editing, Investigation. **Mathis Carrere:** Investigation. **Xiaohua He:** Writing – review & editing, Validation. **Tengchuan Jin:** Writing – review & editing, Validation. **Yixiang Xu:** Writing – review & editing.

## Funding

This work was supported, in part, by the US Department of Agriculture, Agricultural Research Service.

Mention of trade names or commercial products in this publication is solely for the purpose of providing specific information and does not imply recommendation or endorsement by the U.S. Department of Agriculture. USDA is an equal opportunity provider and employer.

## Declaration of competing interest

The authors declare that they have no known competing financial interests or personal relationships that could have appeared to influence the work reported in this paper.

## Data Availability

Data will be made available on request.

## Appendix A Supplementary data

Supplementary material 1 Nb16 coding sequence and plasmid map Supplementary material 1

Supplementary material 2 additional data on food samples Supplementary material 2
