# Supplementary material for: Usage of nanobody-beta-galactosidase fusion in immunoassays and its application in detecting a peanut allergen
Source: Food Chem (Oxf). 2026 Jan 18;12:100357. doi: 10.1016/j.fochms.2026.100357 (PMC12860646; doi:10.1016/j.fochms.2026.100357)

**Usage of nanobody-beta-galactosidase fusion in immunoassays and its application in detecting a peanut allergen**

Yuzhu Zhang ^a^, Shilpa R. Bhardwaj ^a^, Mathis Carrere ^a,b^, Xiaohua He ^a^, Tengchuan Jin ^c^, Yixiang Xu ^a^

^a^ US Department of Agriculture, Agricultural Research Service, Pacific West Area, Western Regional Research Center, 800 Buchanan Street, Albany, CA 94710, USA

^b^ Permanent address: Purpan Engineering School, 75, TOEC route - BP 57611, 31076 Toulouse, France.

^c^ Division of Life Sciences and Medicine, University of Science and Technology of China, Hefei 230027 China

**Supplementary materials**


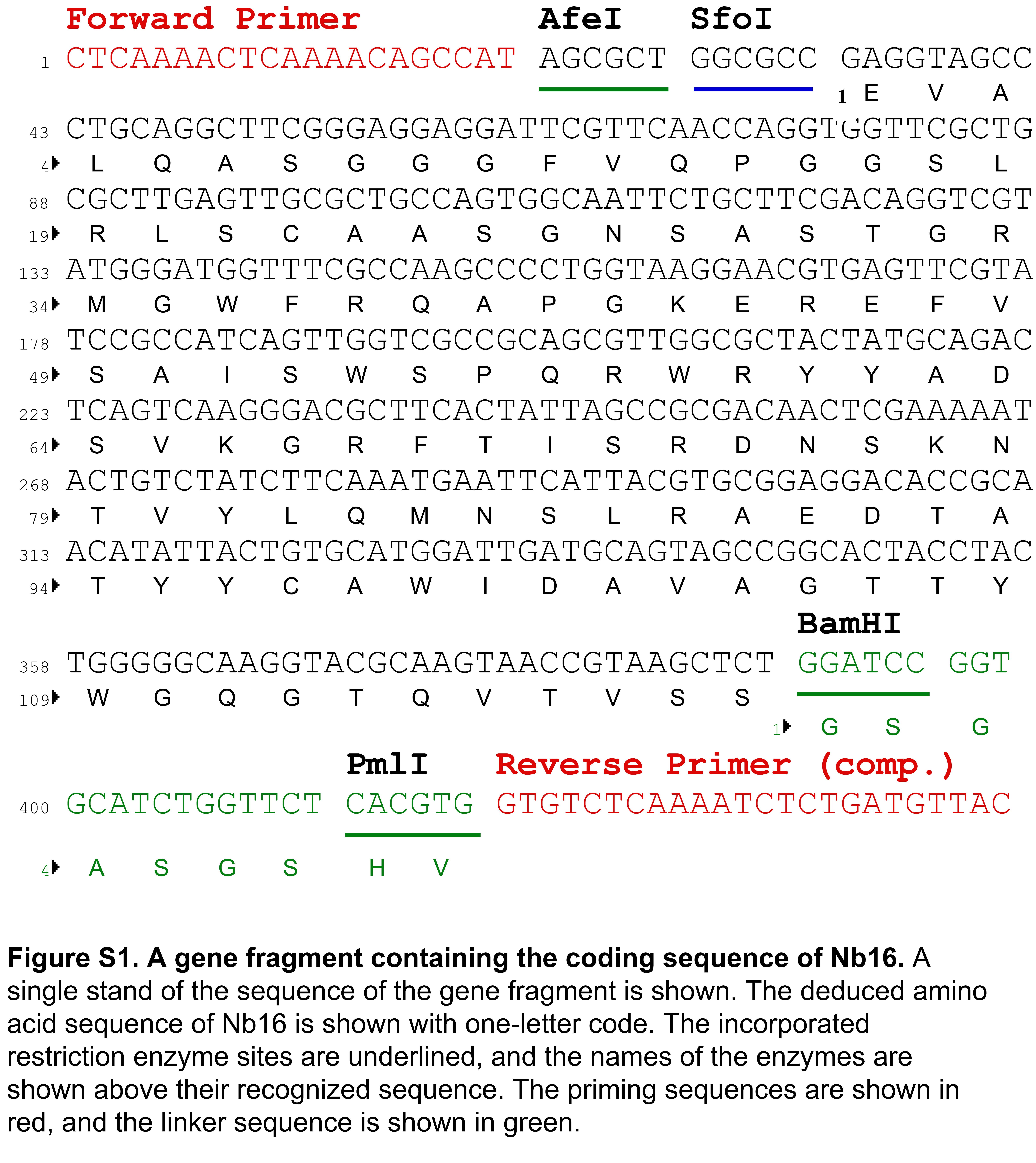


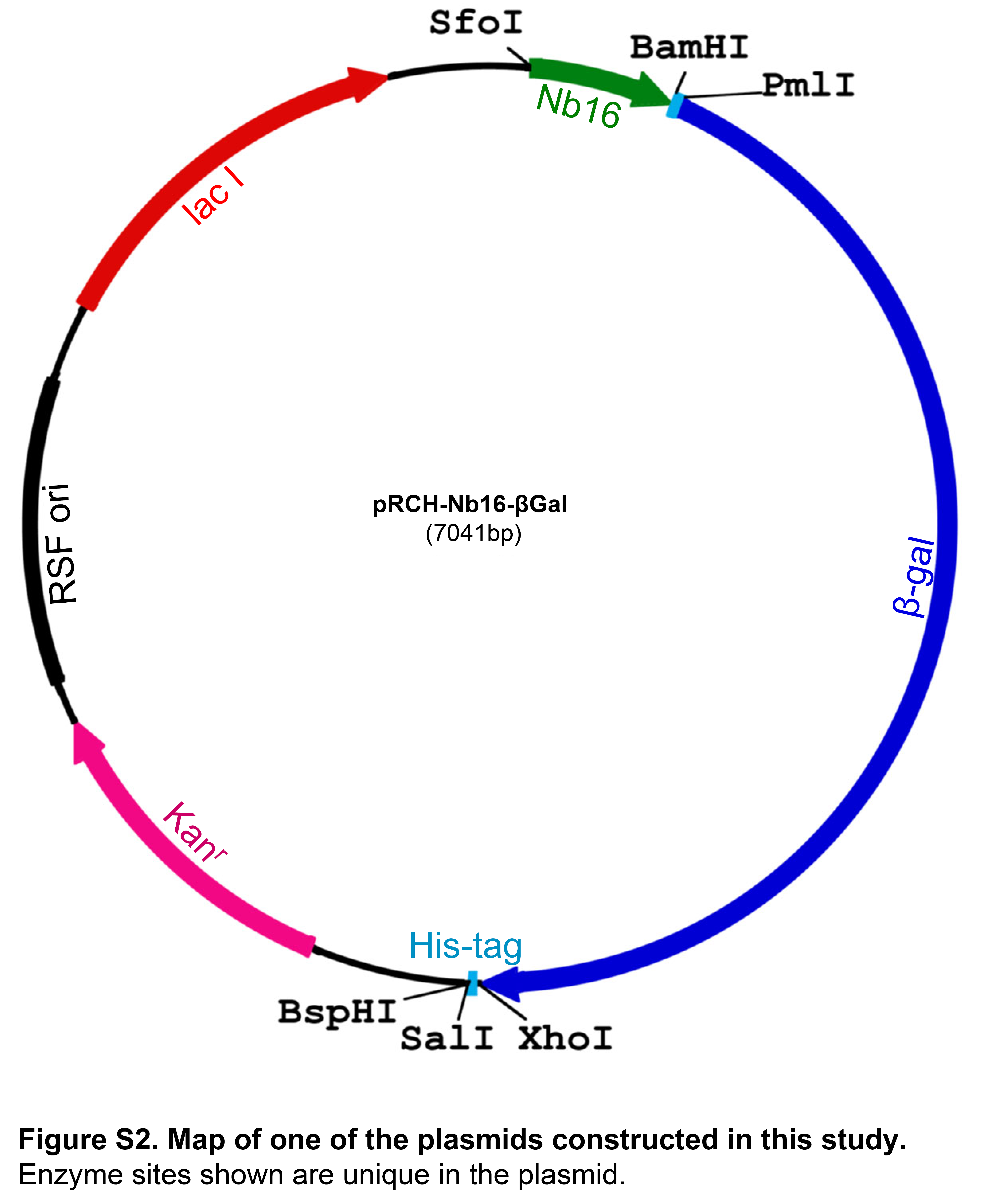

Supplement: Supplementary material 1 — Nb16 coding sequence and plasmid map [file mmc1.docx]
