# Supplementary material for: Usage of nanobody-beta-galactosidase fusion in immunoassays and its application in detecting a peanut allergen
Source: Food Chem (Oxf). 2026 Jan 18;12:100357. doi: 10.1016/j.fochms.2026.100357 (PMC12860646; doi:10.1016/j.fochms.2026.100357)
Supplement: Supplementary material 2 — additional data on food samples [file mmc2.pdf]

# Usage of nanobody-beta-galactosidase fusion in immunoassays and its application in detecting a peanut allergen

Yuzhu Zhang [a], Shilpa R. Bhardwaj [a], Mathis Carrere [a,b], Xiaohua He [a], Tengchuan Jin [c], Yixiang Xu [a]

[a] US Department of Agriculture, Agricultural Research Service, Pacific West Area, Western Regional Research Center, 800 Buchanan Street, Albany, CA 94710, USA

[b] Permanent address: Purpan Engineering School, 75, TOEC route - BP 57611, 31076 Toulouse, France.

[c] Division of Life Sciences and Medicine, University of Science and Technology of China, Hefei 230027 China

## Supplementary data

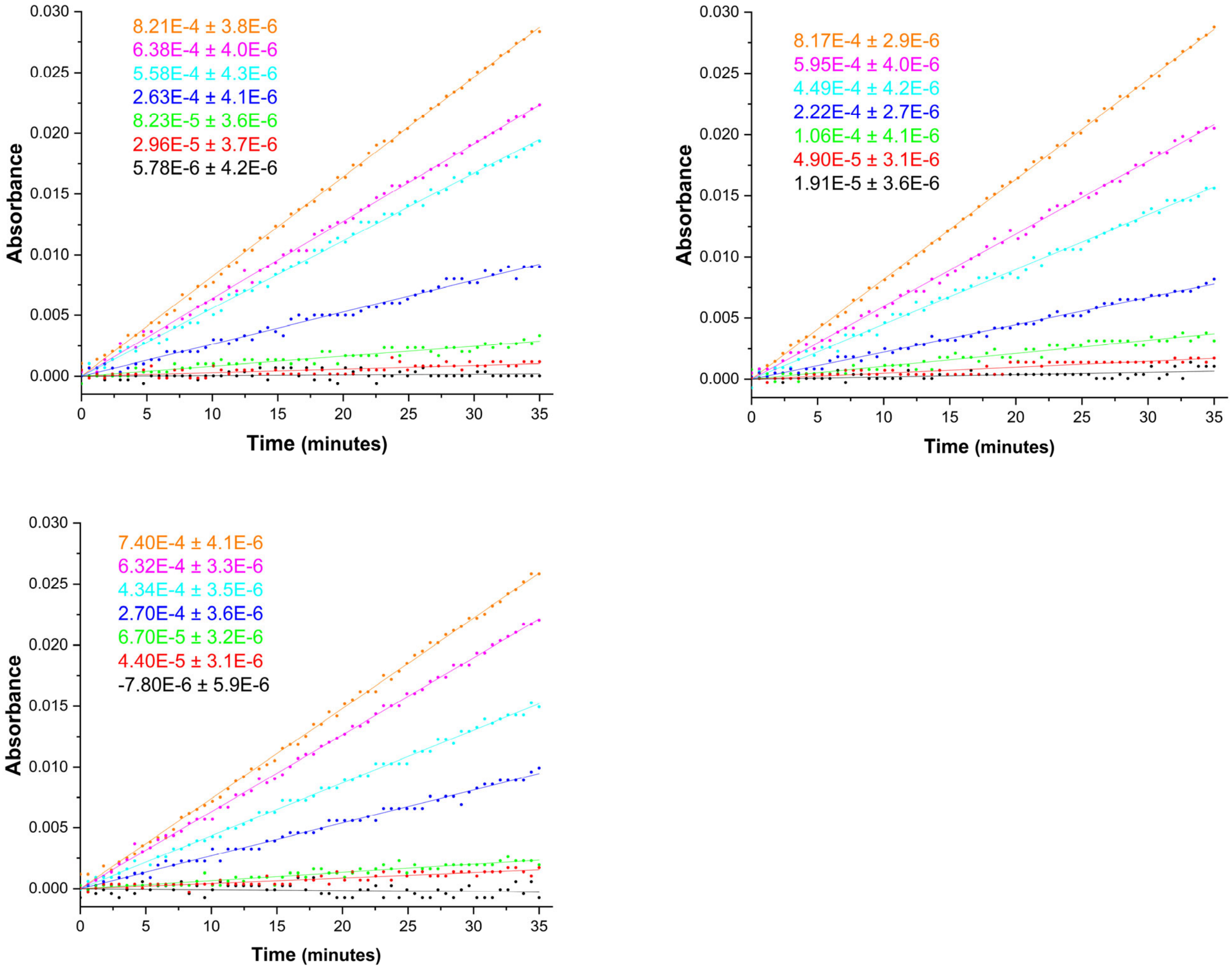


**Figure S3. Direct ELISA detection of peanut proteins in baked food using peanut buffer from manufacturer 1.** Kinetic signal readout during plate incubation with β-gal substrate ONPG. Each panel reports the results of an independent ELISA experiment. In each experiment, each data point is the average of three triplicate wells. Data obtained by coating the plate with diluted muffin extract at peanut protein concentrations of 0.70, 1.39, 3.49, 8.71, 43.32, and 86.65 ppm are shown in red, green, blue, cyan, magenta, and orange, respectively. Data for the negative control, with wells treated with TBS during coating, are shown in black. Linear fits were applied to each data set, and the y-axis intercept of each fit was subtracted from each data point to shift the data set vertically. The straight lines are the results of linear fits of the shifted data. The rate of signal increase (the slope and its standard deviation of the curve fitting) is shown in the insert.

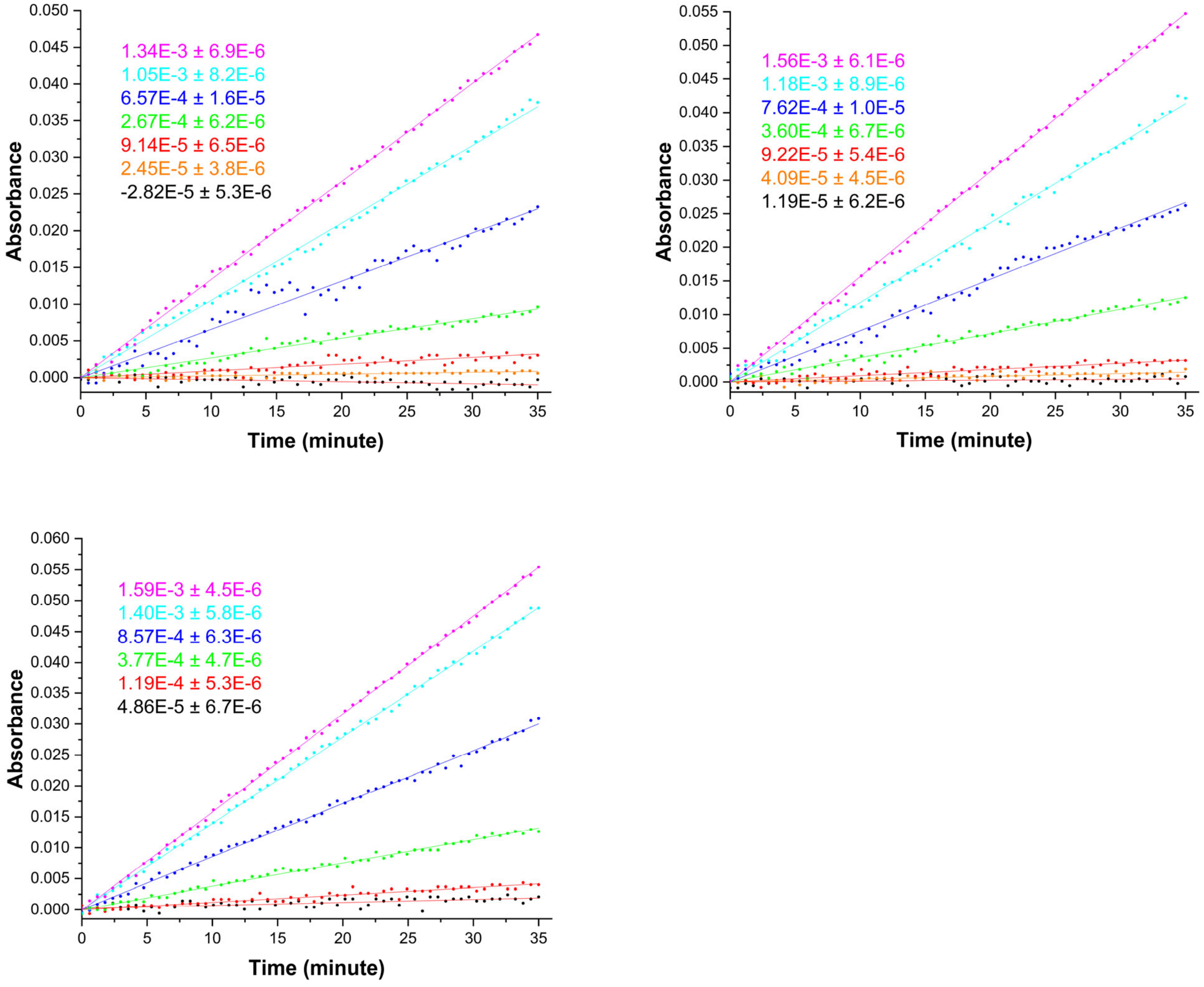


**Figure S4. Direct ELISA detection of peanut proteins in baked food using peanut buffer from manufacturer 2.** Kinetic signal readout during plate incubation with β-gal substrate ONPG. Each panel reports the results of an independent ELISA experiment. In each experiment, each data point is the average of three triplicate wells. Data obtained by coating the plate with diluted muffin extract at peanut protein concentrations of 0.64, 1.28, 3.21, 8.00, 39.80, and 39.60 ppm are shown in orange, red, green, blue, cyan, and magenta, respectively. Data for the negative control, with wells treated with TBS during coating, are shown in black. Linear fits were applied to each data set, and the y-axis intercept of each fit was subtracted from each data point to shift the data set vertically. The straight lines are the results of linear fits of the shifted data. The rate of signal increase (the slope and its standard deviation of the curve fitting) is shown in the insert.

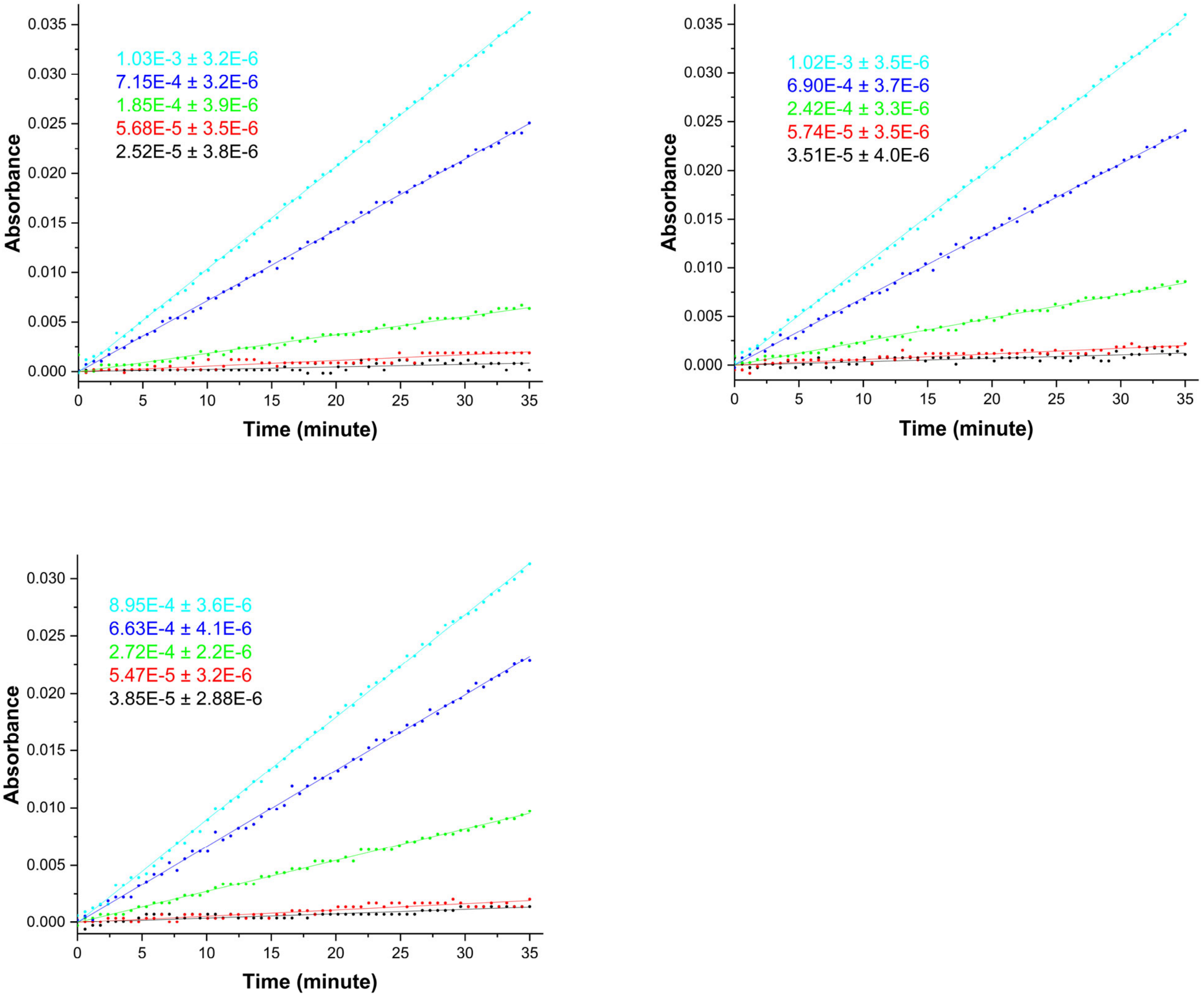


**Figure S5. Direct ELISA detection of peanut proteins in baked food using the peanut buffer from manufacturer 3.** Kinetic signal readout during plate incubation with β-gal substrate ONPG. Each panel reports the results of an independent ELISA experiment. In each experiment, each data point is the average of three triplicate wells. Data obtained by coating the plate with diluted muffin extract at peanut protein concentrations of 1.29, 3.23, 8.05, and 40.05 ppm are shown in red, green, blue, and cyan, respectively. Data for the negative control, with wells treated with TBS during coating, are shown in black. Linear fits were applied to each data set, and the y-axis intercept of each fit was subtracted from each data point to shift the data set vertically. The straight lines are the results of linear fits of the shifted data. The rate of signal increase (the slope and its standard deviation of the curve fitting) is shown in the insert.
